# *Mycoplasma agalactiae* Vaccines: Current Status, Hurdles, and Opportunities Due to Advances in Pathogenicity Studies

**DOI:** 10.3390/vaccines12020156

**Published:** 2024-02-02

**Authors:** Maysa Santos Barbosa, Beatriz Almeida Sampaio, Joachim Spergser, Renate Rosengarten, Lucas Miranda Marques, Rohini Chopra-Dewasthaly

**Affiliations:** 1 Department of Biointeraction, Multidisciplinary Institute of Health, Federal University of Bahia, Vitoria da Conquista 45029-094, Brazil; maysabarbosa_06@hotmail.com (M.S.B.);; 2 Department of Microbiology, State University of Santa Cruz (UESC), Ilheus 45662-900, Brazil; 3 Institute of Microbiology, Department of Pathobiology, University of Veterinary Medicine Vienna, 1210 Wien, Austria; 4 Department of Microbiology, Institute of Biomedical Science, University of São Paulo, Sao Paulo 05508-000, Brazil

**Keywords:** *Mycoplasma agalactiae*, contagious agalactia, vaccine, surface proteins, antigenic variation

## Abstract

Contagious agalactia (CA) is a serious multietiological disease whose classic etiological agent is *Mycoplasma agalactiae* and which causes high morbidity and mortality rates in infected herds. CA is classified as a notifiable disease by the World Organization for Animal Health due to its significant worldwide economic impact on livestock, primarily involving goat and sheep farms. The emergence of atypical symptoms and strains of *M. agalactiae* in wildlife ungulates reestablishes its highly plastic genome and is also of great epidemiological significance. Antimicrobial therapy is the main form of control, although several factors, such as intrinsic antibiotic resistance and the selection of resistant strains, must be considered. Available vaccines are few and mostly inefficient. The virulence and pathogenicity mechanisms of *M. agalactiae* mainly rely on surface molecules that have direct contact with the host. Because of this, they are essential for the development of vaccines. This review highlights the currently available vaccines and their limitations and the development of new vaccine possibilities, especially considering the challenge of antigenic variation and dynamic genome in this microorganism.

## 1. Introduction

Contagious agalactia (CA) was clinically described for the first time in Italy in 1816. Still, it was only in 1923 that *Mycoplasma agalactiae*, one of the main causative agents of CA, was isolated and characterized [1,2]. CA is a multietiological disease with morbidity of up to 100% [3] and sometimes even high mortality of up to 50% in young animals [4], often making it necessary to slaughter the animals to control the disease [2,3]. CA is classified by the World Organization for Animal Health (WOAH, founded as OIE) as a notifiable disease due to its significant economic impact on livestock farming [5]. An estimated loss of over EUR 100,000 was reported on a single farm in Sicily (Italy) with a mixed herd of goats and sheep to control the disease. This indicates that the financial losses related to CA are high and underestimated [6].

According to the OIE [5], in addition to *M. agalactiae*, three other species of mycoplasmas cause a clinically similar disease and, therefore, are also classified as CA-causing agents: *M. capricolum* subsp. *capricolum, M. mycoides* subsp. *capri*, and *M. putrefaciens* [5]. Although *M. agalactiae* is responsible for most of the infections in both goats and sheep [4], coinfections of mycoplasma species have been described [7,8]. *M. agalactiae* has been isolated from infected goats and sheep, including asymptomatic carrier animals, and also from wild animals [7,9,10,11,12,13,14]. Congenital infections with *M. agalactiae* have also been reported [15].

*M. agalactiae* has been reported in several countries, such as Turkey, Iran, Jordan, Canada, the United States, and Brazil, but it has a greater impact in countries along the Mediterranean Sea [1,2]. The presence of *M. agalactiae* in different countries justifies the need for a universal solution to control the disease.

*M. agalactiae* infection in nursing animals is mainly characterized by mammary gland atrophy and decreased or absent milk production, followed by arthritis, conjunctivitis, and abortion; in some cases, pneumonia and pleurisy are also observed. In young animals, pneumonia, arthritis, and blindness stand out. Respiratory and genital problems can also be considered “atypical” signs of the disease [4]. Painful swelling of the joints leads to arthritis, which can progress to loss of motility in chronic disease. Conjunctivitis can progress to causing blindness [2,4]. Less frequently, miscarriage or stillbirth may occur due to inflammation of the uterus [2,16]. Granular vulvovaginitis and testicular inflammation have also been described [2,16]. Systemic spread to distant sites like the spleen, brain, and lungs has been reported in experimental intramammary infection in sheep [17]. Brain lesions in sheep experimentally infected with *M. agalactiae* were described, suggesting a relationship between the presence of this mycoplasma and non-purulent encephalitis and ataxia in young animals [18]. However, the classic symptoms that characterize CA affect the mammary gland, eyes, and joints, although they do not always occur simultaneously. Furthermore, subclinical or asymptomatic forms have been reported, resulting in dissemination and making it difficult to control the agent in the herd [4,14,19,20]. The presence of *M. agalactiae* in semen samples in artificial insemination centers has also been described and indicates the presence of the microorganism in asymptomatic animals in reproductive programs [7,14,20].

Antimicrobial therapy remains the main form of control of CA [21]. However, several factors must be taken into consideration, such as (i) intrinsic resistance to antibiotics such as β-lactams, glycopeptides, and phosphomycin due to the absence of a cell wall; (ii) natural resistance to rifampicin, due to a mutation in the *rpoB* gene; and natural resistance to polymyxin, sulfonamides, and first-generation quinolones due to the absence of lipopolysaccharides and folic acid synthesis. Tetracyclines, macrolides, and pleuromutilin, which inhibit protein synthesis by binding to the 50S or 30S ribosomal subunit, are often used, as are antibiotics that inhibit DNA replication, such as fluoroquinolones [22].

The sensitivity of *M. agalactiae* to different antibiotics has been reported and varies depending on the isolate. Some studies demonstrate the effectiveness of quinolones [23,24,25,26], macrolides [23,26], tetracyclines [23,26], and lincosamides [25] against *M. agalactiae*. However, higher concentrations of antibiotics such as erythromycin [25,26,27], streptomycin [26], chloramphenicol [25], and nalidixic acid [26] are needed to inhibit microbial growth. In samples of *M. agalactiae* obtained over 25 years, there was a decrease in sensitivity to tetracycline and macrolides, except fluoroquinolones [28]. As an alternative antibiotic therapy, the in vitro antimicrobial potential of lactic acid bacteria present in the mammary glands of small ruminants against *M. agalactiae* was also evaluated and suggested as a measure to control or prevent mastitis caused by the microorganism [29]. Therefore, with the disadvantages of antimicrobial therapy such as the selection of resistant strains, changes in milk quality, quarantine period, dissemination of the microorganism during treatment, and costs related to control, more effective prevention approaches are necessary. Here, we review the current status of and research on *M. agalactiae* vaccines and address advances in studies on its virulence and pathogenicity to correlate their contributions towards developing more efficient vaccines.

## 2. Mycoplasma Agalactiae Vaccines

### 2.1. Commercial Vaccines

Commercial vaccines to control infections caused by *M. agalactiae* are available in Europe and Asia [30]. Most commercial vaccines are inactivated, although attenuated vaccines provide better immunity [30,31]. For clarity, this section is further divided into three subparts.

#### 2.1.1. Attenuated Commercial Vaccines

Live attenuated vaccines against *M. agalactiae* have been described as one of the most protective vaccines as they promote a lower risk of the animal being affected by any clinical sign of CA in addition to reducing the excretion of mycoplasmas in milk, reducing the spread of infection in the herd [32]. However, reversion of the virulence of the vaccine strain may occur. These vaccines can also promote a temporary infection and for this reason, they are not recommended for lactating animals [5]. Though not permitted in many European countries, an attenuated *M. agalactiae* vaccine is commercialized in Turkey [5,33].

The AIK vaccine strain from Turkey was initially attenuated for 40 passages, followed by another 30 passages to reduce its pathogenicity, although this also reduced its protection [34]. Vaccination of goats in Turkey with this attenuated AIK vaccine strain proved effective. Although the vaccine conferred 85% immunity, a humoral response was not detected, probably due to the loss of virulence genes like the *nif* locus or due to antigenic diversity between the vaccine strain and the ELISA kit used for analysis [34]. Similarly, another study did not identify a humoral immune response induction after immunization with an attenuated vaccine [32].

Although attenuated vaccines may promote some temporary clinical signs, they are more efficacious, cheaper, and can be protective in local outbreaks if the vaccine is administered around the same time [32]. However, experimental results demonstrate some limitations, such as the absence of other correlates of protection besides antibodies in vaccinations against *M. agalactiae* and the implication of the antigenic diversity of this microorganism. Serological tests with greater sensitivity to different strains are needed. In addition, although it is known that strains lacking the *nif* locus are avirulent and do not produce an IgG immune response [32,34], genetic analyses have not confirmed the absence of the *nif* locus in the AIK strain.

#### 2.1.2. Inactivated Commercial Vaccines

Although inactivated vaccines are less protective, they do not have the disadvantages of attenuated vaccines and are therefore the most widely commercialized vaccines against *M. agalactiae*. Inactivated vaccines are permitted in most countries [30]. In Spain, the available commercial vaccines containing inactivated *M. agalactiae* are as follows: Algontex (CZ Veterinaria S.A., Porriño, Spain), composed of strain N-262 and adjuvants Marcol 52, Montanide 103, Montane 80, and Polysorbate 80; Agalaxipra^®^ (Laboratorios HIPRA, S.A., Amer, Spain), containing inactivated *M. agalactiae* strain 784 and aluminum hydroxide as an adjuvant; Myo Galax (Laboratorios Ovejero S.A., León, Spain), containing the Ag 8 strain with aluminum hydroxide, aluminum sulfate, and potassium dodecahydrate; and Agalax UNO (Laboratorios Syva, S.A., Madrid, Spain), containing strain N84 in aluminum hydroxide (also available in Cyprus, Greece, and Saudi Arabia) [30]. In Romania, Agavac is available, containing an injectable suspension of the Ag 6 strain inactivated in formaldehyde and with added aluminum hydroxide [35]. In Italy, commercialized vaccines include Ovax agalassia (Fatro Industria Farmaceutica Veterinaria S.p.A., Italy) with saponin as an adjuvant and Aglovax (MSD Animal Health S.r.l., Italy), composed of *M. agalactiae*, *M. capricolum* subsp. *capricolum,* and *Mycoplasma mycoides* subsp. *capri* with aluminum hydroxide. In Iran, the Agalactivac oil/Laxydoll oil vaccine (Vetal/ Dolivet, Turkey), containing the inactivated AIK strain, is sold [30]. However, there are few published data in scientific articles regarding inactivated commercial vaccines.

#### 2.1.3. Autogenous Vaccines

Due to CA being endemic in Italy, autogenous *M. agalactiae* vaccines of unproven efficacy, obtained from homogenates of milk, brain, and mammary glands from infected sheep, were used for a long time. However, the use of this type of vaccine was discontinued due to its relationship with a severe scrapie outbreak in 1997 and 1998 [36]. In a prospective study, it was observed that provinces where the CA vaccine potentially contaminated with scrapie had been administered had higher rates of scrapie outbreaks when compared to the provinces where the vaccine had not been administered [37]. In Italy, an autogenous vaccine, combined with aluminum hydroxide and regulated by D.M. 3/17/94 No. 287, is inactivated by formalin, phenol, or saponin [30]. However, a more robust immune response consisting of higher levels of antibodies as well as leukocytes, neutrophils, and blood platelets was evident with a bivalent autogenous vaccine (*M. agalactiae–Staphylococcus aureus*) administered with aluminum hydroxide [38]. This vaccine proved safe and efficient, but was only evaluated for 17 weeks in ewes [38].

Autogenous vaccines, which have efficacy depending on the phase and intensity of the outbreak at the time of vaccination, represent a convenient and practical option for authorized and commercialized products. However, they offer solutions for that particular location, as their application is recommended only on farms with confirmed outbreaks [30]. A significant disadvantage of autogenous vaccines is the large quantity of bacteria required (10^8^–10^9^ CFU/mL) [30,38].

### 2.2. Experimental Vaccines

Table 1 provides an overview of some of the most important experimental M. agalactiae vaccines, including inactivated vaccines, DNA vaccines, and subunit vaccine candidates, as also discussed below.

#### 2.2.1. Inactivated Vaccines

Inactivated vaccines against CA are the most studied as they do not have the disadvantages of attenuated vaccines. Because they promote a lower immune response, vaccine formulations with different adjuvants and inactivation methods have been studied. However, it is essential to consider that the formulation potential of each immunization will depend on its intrinsic characteristics [39].

Saponin-inactivated vaccines showed better results in protecting against clinical signs than those inactivated by formalin [39,40,41]. Additionally, developing an immune response with activation of T cells and IFN-gamma was correlated with developing protection against the disease [39]. Phenol-inactivated vaccines have also been shown to be protective against CA caused by *M. agalactiae* [41] and *M. mycoides* subsp. *capri* [42]. On the other hand, in mice, formalin-inactivated vaccines were more efficient when using the adjuvant polyinosinic–polycytidylic acid by promoting a Th1-mediated response [43]. However, there are no data on using these adjuvants in experimental vaccinations against *M. agalactiae* in goats or sheep.

**Table 1:** Overview of experimental vaccines against *Mycoplasma agalactiae*.

Strain/Antigen	Vaccine Type	Vector/Adjuvant	Host	Route Number of Doses Dosage	Duration of Immunity	Animals Challenged	Reference
*M. agalactiae* NU-658	Phenol-inactivated	Aluminum hydroxide	Sheep	s.c. 3 doses 10^9^ CCU/mL	11 months	Yes	[41]
	Formalin-inactivated	-					
	Heat-inactivated	Aluminum hydroxide					
	Sodium hypochlorite-inactivated	Aluminum hydroxide					
	Saponin-inactivated	-					
*M. agalactiae* L9, IN3, 9B + *M. mycoides* subsp. *capri* AG1, 153/93, LCIN3	Formalin-inactivated	Aluminum hydroxide Aluminum hydroxide + Quil A^®^ (Superfos A/S, Vedbaek, Denmark))	Goats	s.c. 2–3 doses >5 × 10^10^ CFU/mL (each strain)	1 year	Yes	[42]
		-					
*M. agalactiae* Ag6	Formalin-inactivated	Freund’s complete adjuvant	Mice	i.p. 1 dose 10^9^ cells	12 days	- ^§^	[43]
		Freund’s incomplete adjuvant					
		Lipopolysaccharide					
		Quil A^®^					
		* poly I:C					
		** poly A:U					
		LiCl					
		Calcium phosphate gel					
*M. agalactiae* P20BrPB03	Formalin-inactivated	Aluminum hydroxide	Goats + sheep	s.c. 2 doses 5 mg of protein per dose		Yes	[44]
		Montanide IMS 2215 VG			171 days		
		Montanide Gel 01					
*M. agalactiae* Ba/2	Beta-propiolactone-inactivated	Montanide ISA 563	Sheep	i.t. 2 doses 2 × 10^9^ DNA copies/mL		Yes	[45]
		Montanide ISA 563, Marcol 52, Montane 80 (50:45:5 ratio)			8 weeks		
		Montanide ISA 563, Marcol 52, Montane 80 (30:63:7 ratio)					
*M. agalactiae* Ba/2	Beta-propiolactone-inactivated	Montanide ISA 563, Marcol 52, Montane 80 (30:63:7 ratio)	Sheep	i.t. 2 doses 2 × 10^9^ DNA copies/mL	11 months	Yes	[46]
*M. agalactiae*	Phenol-inactivated (autogenous)	Aluminum hydroxide	Sheep	s.c. 2 doses 10^9^ CFU/mL	16 weeks	No	[38]
*M. agalactiae* + *Staphylococcus aureus*							
*M. agalactiae* L9, AGIN3, 9B + *M. mycoides* subsp. *capri* AG1, 153/93, IN3	Formalin-inactivated	Aluminum hydroxide + Quil A^®^	Goats	s.c. 2 doses >5 × 10^10^ CFU/mL (each strain)	7 months	No	[40]
	Phenol-inactivated	Aluminum hydroxide + Quil A^®^					
*M. agalactiae*	Inactivated	Aluminum hydroxide Mineral oil	Goats	3 doses	6 months	No	[47]
P48	DNA	pVAX1/P48	Mice	i.m. 3 doses 50 µg (1 µg/µL)	8 weeks	- ^§^	[48]
*M. agalactiae*	Formalin-inactivated (therapeutic)	Quil A^®^	Sheep	s.c. 2 doses	-	No	[49]
^†^ MAG_1560 MAG_6130 P40	Recombinant subunit	Freund’s adjuvant	Rabbits	i.m. 3 doses 500 µg	42 days	- ^§^	[50]

* poly I:C (polyinosinic–polycytidylic acid); ** poly A:U (polyadenylic–polyuridylic acid). (s.c.) subcutaneous immunization; (i.p.) intraperitoneal immunization; (i.t.) intratail immunization; (i.m.) intramuscular immunization. ^§^ Challenge model not available. ^†^ Immunization in goats in progress.

In yet another attempt, vaccines emulsified in mineral oils as adjuvants demonstrated better results when using the composition of Montanide ISA-563 (Seppic Inc., Paris, France), Marcol-52 (Esso Italiana S.r.l., Rome, Italy), and Montane-80 (Seppic Inc., Paris, France) (ratio 30%, 63%, and 7%, respectively), which were protective and prevented the development of clinical signs and infection with *M. agalactiae* [45]. Although vaccines containing mineral oil-based adjuvants demonstrate the ability to generate high levels of antibodies for at least 8 months after vaccination and have high immunogenicity, it is essential to note that there is the possibility of triggering a granulomatous reaction at the site of inoculation [46].

In another comparative study using four different vaccines, the best results were observed with the attenuated vaccine marketed in Turkey but forbidden in Europe, followed by a saponin-inactivated vaccine, an autogenous vaccine inactivated by formalin and containing saponin, and a commercial vaccine inactivated by formalin and using aluminum hydroxide as an adjuvant [32]. Amongst these, the least effective protection results were demonstrated by the inactivated commercial vaccine [32]. The saponin-inactivated vaccine and the autogenous vaccine inactivated by formalin and containing saponin demonstrated the activation of CD4^+^ memory Interferon-γ^+^ T cells from the seventh day of infection to the tenth, suggesting that the timing of expansion of this subset could be considered as a correlate of protection [39].

During a preliminary study in Italy, a flock of sheep suffering from mastitis, keratoconjunctivitis, and arthritis failed to respond to antimicrobial treatment and were therapeutically vaccinated [49]. The formalin-inactivated autogenous vaccine improved clinical signs and decreased the excretion of *M. agalactiae* in milk [49]. However, despite these positive results, other studies must be conducted to assess whether the vaccine was protective.

Inactivated vaccines are an alternative to attenuated vaccines but do not promote a completely protective immune response and have variable results depending on the adjuvant of choice or the inactivation method (Table 1). Furthermore, due to the very high antigenic variability of *M. agalactiae*, vaccine strains may not be broadly protective against local strains. The results point towards the need for further studies to establish new correlates of protection based on the cellular immune response and formulations that promote lasting immunity.

#### 2.2.2. Nucleic Acid-Based Vaccines

Another alternative strategy is the DNA vaccine formulated from the gene encoding the immunodominant P48 lipoprotein. Although it has been shown to induce Th1 and Th2 immune responses in BALB/c mice, it has not been evaluated in sheep or goats [48]. By promoting humoral and cellular immune responses, vaccines based on nucleic acids have been evaluated in small ruminants demonstrating promising results against bluetongue virus, rift valley fever virus, lentiviruses [51], *Haemonchus contortus* [52], and *Toxoplasma gondii* [53].

#### 2.2.3. Subunit Vaccine Candidates

With the availability of the gene sequence of several *M. agalactiae* strains in recent years, some proteins have been identified and studied as possible vaccine candidates. Several *M. agalactiae* proteins have been shown to have high immunogenicity and immunoreactivity in addition to promoting a humoral immune response, such as proteins P40 [54], P30 [55], P48 [56,57], P80 [58], MAG_1000, MAG_2220, MAG_1980, PhnD, MAG_4740 and MAG_2430 [59], MAG_5040 [60], and Vpmas [59,61,62]. More recently, MAG_1560 and MAG_6130 proteins were identified by our group as novel antigenic proteins using bioinformatic analyses and confirmed to be immunogenic as they recognized sera from infected goats and sheep [50]. In addition, MAG_6130 was demonstrated to play important roles in host colonization and pathogenicity via its adherence functions [63].

Some of these molecules are not recommended as vaccine candidates, either due to their high-frequency antigenic variations or due to the lack of expression in some isolates. Furthermore, no study has evaluated the effectiveness of these molecules in preventing infection or reducing clinical signs. It is expected that new antigens capable of activating B and T cells will be proposed using reverse vaccinology with the availability of more sequenced genomes of *M. agalactiae* strains. Studies with multivalent or multiepitope immunizations with selected epitopes to stimulate humoral and cellular responses followed by host challenges and analyses of other correlates of protection, in addition to antibodies, could provide good vaccine candidates.

## 3. Hurdles, Challenges, and Opportunities in Developing Next-Generation *M. agalactiae* Vaccines

Despite the promising results of attenuated vaccines, their use is not permitted in all countries [30,32]. On the other hand, inactivated vaccines have variable results and promote a short-term memory response [33]. Different categories of experimental vaccines are under development, but the results are still preliminary and further studies of immunization in the host are lacking.

### 3.1. Challenge Model

A reproducible and appropriate challenge model is essential for developing a good vaccine; this is especially critical for mycoplasmas, as they show strict host and tissue specificity. The lack of suitable small animal models is one of the main reasons why, in spite of a large number of identified immunodominant proteins, there is still a lack of visible progress in using these molecules as vaccines. Although there are established models of conjunctival [64] and intramammary *M. agalactiae* infections in the natural host, i.e., in sheep [19,65], it is difficult, time-consuming, and expensive to conduct experiments using small ruminants due to the many logistic problems associated with large animal trials. The development of sophisticated 3D polarized coculture systems from existing primary cell in vitro models would definitely expedite initial screening steps [66], though a robust small animal model is imperative for challenge experiments. The limited availability of kits to assess immunological responses in sheep and goats also limits proper analyses and evaluations. Experimental challenges with large groups, especially with lactating animals and in restricted areas, are serious limitations. Few challenge models have been described, for instance, keeping vaccinated animals in contact with animals from herds where the presence of *M. agalactiae* had previously been detected [42], or immersing the animals’ udders in a solution containing a bacterial culture [44], or via the intramammary [41] and nasal routes [45]. However, the absence of a smaller animal model and a dearth of specific kits to evaluate the immune responses of goats and sheep are a big hindrance and delay the development of new vaccine alternatives.

### 3.2. Comprehending Highly Dynamic Antigenic Surface and Complex Pathogenicity Traits of M. agalactiae

**Figure 1:**
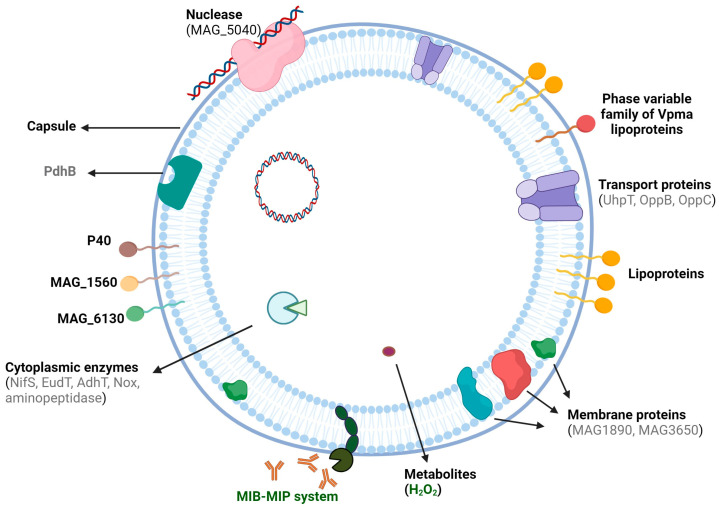
A schematic overview of the potential pathogenicity determinants of *Mycoplasma agalactiae* that could serve as specific antigen targets for vaccine development. The majority of these factors are surface-localized, and some, like PdhB, seem to “moonlight” on the cell surface for host interactions. Although, as discussed in the text, most of these proteins have been shown to be involved in important pathogenicity-related phenotypes, the ones highlighted in green are yet to be proven as pathogenicity factors by experimental studies, whereas those in grey correspond to mutants attenuated in sheep infection trials where pools of transposon mutants were screened via negative selection methodology. [Created with BioRender.com].

Another challenge in developing broadly effective vaccines arises from the cell surface complexity and variability observed in antigenic proteins of *M. agalactiae*. In mycoplasmas, many of these surface molecules that are exposed to the environment are important for host cell interactions, making them important vaccine targets. Figure 1 provides a schematic overview of important antigenic proteins as well as identified pathogenicity determinants of *M. agalactiae* that could serve as promising vaccine candidates. Extensive analyses of 245 field isolates originating from different countries allowed for the serological grouping of this pathogen into eight serotypes (A–H), the majority of which (79.3%) were classified as belonging to serogroup A, and four serotypes exhibited high phenotypic variation on their surface [67]. Another study reported high antigenic variability amongst French strains compared to vaccine strain 190 and the international-type strain PG2. Although no particular differences were observed between the virulent and the “supposedly” avirulent strains, antigenic variation was related to geographic origins and differences within strains from the same group were also indicated [68]. Subsequently, proteome analyses identified that the PG2 strain and two field isolates from an Italian island have differences in protein expression and allowed for the identification of 194 surface proteins [69]. Together, these results demonstrate the enormous antigenic variability of isolates of this species in different regions and the need for more studies to identify the core antigens of *M. agalactiae* common in strains from several countries, enabling the development of a vaccine with broad protection.

Specifically, among the molecules involved in antigenic variation in *M. agalactiae*, Vpmas are the most studied. Vpma lipoproteins (variable proteins of *M. agalactiae*, also called Avg—Agalactiae variable gene) [61,70] stand out as abundantly expressed highly immunogenic proteins of *M. agalactiae* that undergo phase variations at a very high frequency due to DNA rearrangements within the Vpma multigene locus [61,70,71,72,73]. The *vpma* locus promotes broad antigenic diversity even within a single strain. Although six genes are found in the single *vpma* locus (*vpmaU, vpmaV, vpmaW, vpmaX, vpmaY,* and *vpmaZ*) of type strain PG2 [73], strain 5632 has 23 *vpma* genes organized in two loci, locus I (16 genes) and locus II (7 genes), which allow for the concomitant expression of two Vpmas, multiplying the number of possible combinations and permutations in these antigenic surface molecules [72]. In contrast, even though two *vpma* loci are seen in the genome of the Greek strain GrTh01 (locus I contains only the *vpmaW*, *vpmaX,* and *vpmaZ* genes and locus II only *vpmaY*), they are highly degenerated and Vpma expression is rather doubtful considering the current genomic information [74]. In another study phenotypically analyzing strain GM139, it was observed that there is a predominant expression of VpmaV (which is relatively less expressed in PG2); however, there are no data on how the *vpma* locus is organized in GM139 [75]. By varying its antigenicity, *M. agalactiae* can avoid host immune responses more efficiently, thus posing limitations in the development of vaccines.

Immunodominant antigens essential for virulence in most pathogens are under pressure to evade the host’s immune system [76]. The Xer1 recombinase mediates DNA rearrangements at the *vpma* gene locus to express one *vpma* gene while others are silenced; however, this mechanism has additional host-mediated regulation through immunological pressure [62,77]. Anti-Vpma antibodies cause repression of the target Vpma and induce the expression of another molecule from the *vpma* locus even if Xer1 is inactivated [77]. VpmaY and VpmaU are regarded as representatives of two homologous groups of Vpma based on the N-terminal sequences and other shared sequences [65,73]. In experimental sheep infections, Vpma “phase-locked” mutants (PLMs) stably expressing either only the VpmaU protein or the VpmaY protein were shown to induce reduced immunogenicity and less ability to spread to and invade the udder compared to the Vpma phase-variable wild-type strain PG2 [65]. In these conjunctival and intramammary cochallenge studies with PLMs expressing only VpmaY or VpmaU, a predominance of mutants expressing only VpmaY was demonstrated, thereby indicating differences in the in vivo fitness and pathogenicity potential of Vpma proteins [62].

When all six isogenic expression variants of the PG2 Vpma multigene family were individually tested in sheep intramammary infections using six different PLMs, the results demonstrated that the inability to alter the Vpma protein does not prevent the initiation of mastitis for individual PLMs, but PLMU expressing VpmaU clearly showed a defect in host colonization and multiplication for the first 24 h, confirming the results of the previous cochallenge study [78]. Additionally, the study indicated a higher potential for the systemic spread for mutants expressing VpmaV and VpmaX, reiterating the significance and differential pathogenicity of Vpmas [78].

Altogether, with Vpmas being one of the most abundant immunodominant lipoproteins, these studies not only highlight the importance of phase-locked mutants (PLMs) in advancing our knowledge about Vpmas and their role in *M. agalactiae’s* pathogenicity and persistence, but also offer a possibility to exploit them further for prophylactic strategies [79]. Although the differences in antigenic profiles between isolates may be an obstacle in the development of globally effective vaccines, all information related to Vpmas, especially also considering their novel role as important cytadhesins [80], and their involvement in serum killing [81], might suggest new directions for vaccine development with more thorough studies involving Vpmas. Although antigenic variation proteins are generally not considered ideal vaccine candidates, the construction of multiepitope antigens can solve this limitation.

Innovative strategies for vaccine design must focus on identifying antigens that remain unexposed to immune pressure during infection and should not trigger elevated immune responses during the disease [76]. In this context, when evaluating PG2 mutants by negative selection in sheep during experimental intramammary infection, mutants that possessed transposon insertions in genes MAG1050, MAG2540, MAG3390, *uhpT*, *eutD*, and MAG4460 were unable to colonize the udder and lymph nodes [82]. Additional genes, including *pdhB*, *oppC*, *oppB*, *gtsB*, MAG1890, MAG5520, and MAG3650, were required for spreading to distant sites such as the spleen, liver, lungs, uterus, kidneys, synovial, brain, heart, and carpal joint tissues, demonstrating that these genes are involved in the dissemination of *M. agalactiae* [83]. These in vivo studies in the natural host of *M. agalactiae* have provided invaluable information about the genetic loci involved in its pathogenicity and systemic spread. Each of these mutants and their corresponding genes need to be individually and elaborately studied and tested for their involvement and contribution to *M. agalactiae’s* disease progression. However, the lack of a smaller animal model, and partially also because of the recalcitrance of mycoplasmas to targeted genetic manipulation, evaluating each gene in isolation is difficult. If it provides sufficient protection and cannot revert to virulence in vivo, a mutant strain lacking these genes could provide a successful alternit ative attenuated vaccine.

In addition to protein antigenic variation, the secretion and high-frequency phase variation of the polysaccharide β-(1→6)-glucan by most *M. agalactiae* isolates has also been observed [84]. The presence of this polysaccharide led to the unusual killing of the mycoplasma cells in goat serum, thereby controlling its serum susceptibility, which can play an important role in dissemination within the host. In addition to *M. agalactiae*, the presence of this polysaccharide has also been demonstrated in *M. mycoides* subsp. *capri* [84]. As this capsular component is an important virulence factor in this species, attenuated strains of *M. mycoides* subsp. *capri* lacking galactofuranose have been described [85]. However, immunizations with these attenuated *M. mycoides* subsp. *capri* strains lacking galactofuranose did not induce protective immunity [85]. Although further studies are needed to evaluate *M. agalactiae* capsule regulation in vivo, these results should be considered in developing glycoconjugate vaccines. Promising results have been described with glycoconjugate vaccines against *M. mycoydes* subsp. *mycoides* infections [86,87].

Other immunodominant proteins, namely P48 [56,57,88], P30 [55], P40 [54], P80 [58], and nuclease MAG_5040 [60], have also been identified in *M. agalactiae* by several independent researchers. An immunoproteomic study not only confirmed some of the earlier molecules, but also identified new molecules capable of stimulating a humoral response, namely P48, P80, MAG_1000, Vpma, and MAG_1000, MAG_2220, MAG_1980, phnD, MAG_4740, and MAG_2430 [59]. In addition to inducing the production of antibodies, three of these molecules, P48, P80, and MAG_1000, were also capable of generating NETosis, producing IL-8, and activating TLR-2 in sheep neutrophils [89]. Recently, we identified P40 and two novel proteins, MAG_1560 and MAG_6130, as immunogenic proteins using immunoinformatics and validatory experimental studies [50]. Although host antibodies recognize these molecules, it has yet to be evaluated whether they protect the host against infection by *M. agalactiae*. Some of these proteins are not present in all isolates. Still, their use in a multiepitope/multivalent subunit vaccine is a promising possibility as long as the addition of too many antigens is not a commercial disadvantage.

The adhesion of bacteria to a host cell is the first stage of colonization and is an essential step in establishing infection [90,91]. For vaccinology, adhesins are promissory molecules because they are required for infection and are exposed to the surface to be accessible to the immune system [92,93]. To date, *M. agalactiae* proteins that have demonstrated the ability to adhere to the host cells are P40, in lamb joint synovial cells [54]; Vpmas (exhibiting differential adherence, with VpmaV and VpmaU being the most and least adherent variants, respectively), in HeLa and mammary stromal and endothelial cells [80]; and also MAG_1560, in HeLa cells and mammary stromal cells [63]. In this context, cues could be taken from several reported vaccine strategies that used bacterial adhesins as vaccine targets [94,95,96,97,98,99,100,101,102,103,104], which could also be an alternative to avoid CA caused by *M. agalactiae*.

The first molecule reported to be involved in the invasion of *M. agalactiae* into eukaryotic cells (HeLa) was the B subunit of pyruvate dehydrogenase [105]. Mutant strains for this molecule demonstrated less invasiveness in HeLa when compared to the wild-type and complementary strain [105]. In agreement with this previous study, the *pdhB* mutant could not disseminate to distant host sites compared to the wild-type strain during an experimental intramammary sheep infection. Although the exact role of this protein in invasion or infection is unknown, a novel chimeric recombinant protein PDHB-P80 has been reported as a potential diagnostic tool [106], further highlighting its importance. In addition to PdhB, Vpmas have also been demonstrated to play a role in the invasive capacity of *M. agalactiae*, mainly the variant expressing VpmaV [80].

The adhesion and invasion of the pathogen to the cell requires a stable association and binding capacity to other host molecules [107]. MAG_6130 and P40 cytoadhesin proteins bind to extracellular matrix molecules such as fibronectin and lactoferrin. Additionally, P40, MAG_6130, and MAG_1560 also bind to fibrinogen at different levels of affinity, and unlike MAG_6130 and MAG_1560, P40 binds to plasminogen [63]. All these characteristics could be critical when considering these potential vaccine candidates.

Stimulating the production of antiadhesin/invasin antibodies can prevent or reduce the colonization of microorganisms [108]. However, receptors involved in the adhesion and invasion of *M. agalactiae* have yet to be studied and could also represent an alternative in the development of anti-infection or prevention strategies.

### 3.3. Understanding Immune Responses

The factors that lead to the persistence of mycoplasma in host tissues exhibiting strong immunoinflammatory responses are not yet clear [109,110]. Understanding the induced immune response and the strategies used by this pathogen to thrive in an immunocompetent host is important to support the development of new efficacious vaccines. After an initial innate immune response that is ineffective in reducing pathogen counts, a relatively short-lived specific humoral response is induced in about a week during which high titers of *M. agalactiae*-specific antibodies are known to coincide with reduced mycoplasma excretion in milk [109]. Though protective, the humoral immune response is limited and unable to get rid of the pathogen, leading to chronic infections. Not only this, the interaction of the cellular immune response via the lymphoplasmacytic reaction is also well established [109]. Elevated numbers of all subsets of specific immune responses, for instance, those corresponding to MHC-II, IgG, IgA, CD3, CD4, and CD8, have been observed during the subacute stage of infection. However, these results did not correlate with the antibody response in blood; they demonstrated a reduced CD4/CD8 ratio (102). All this highlights the need to activate cellular and humoral immunity during the process of a protective response. The lack of knowledge of exactly which arm of immunity is protective is a hindrance, and filling this knowledge gap would help to design a rational next-generation vaccine. Also associated with vaccine efficacy, microorganism excretion in body secretions is a crucial tool in determining the risk of infection spread [32]. Despite stimulating humoral and cellular responses, inactivated and attenuated vaccines present limitations due to the duration of protection [32,34,39,45,46]. Experiments indicate that different adjuvant compositions in formalin-inactivated *M. agalactiae* vaccines can modulate the immune response in Th1 or Th2 profiles [43]. In this context, nucleic acid-based vaccines can be developed to include immunomodulatory genes of the immune response, such as cytokines, chemokines, and costimulatory molecules [111,112]. Similarly, subunit vaccines could also be designed to contain epitopes that modulate B and T cells [113,114,115].

## 4. Conclusions

In summary, *M. agalactiae* infections cause serious socio-economic losses in regions where goat and sheep farming are important. Due to the disadvantages of antimicrobial therapy, little protection provided by inactivated vaccines, and the promotion of a temporary infection caused by attenuated vaccines, it is necessary to develop new and more efficient vaccine strategies to prevent *M. agalactiae* infections. Studies that explore new vaccine targets while moving away from traditional models (inactivated and attenuated) are still limited. The expectation is that advances in understanding the virulence and pathogenicity factors of *M. agalactiae* in different isolates, combined with new antigen selection and delivery techniques as well as the identification of new adjuvants, may facilitate the development of promising vaccine candidates. Multivalent/multiepitope vaccines with antigens that stimulate humoral and cellular responses and/or genetically attenuated vaccines should be considered in future studies. These candidates would not only be commercially viable, but also safe, effective, and accessible on a global scale.

## Data Availability

Data sharing not applicable.
